# Digitale Assistenzsysteme zur Unterstützung der Alltagsbewältigung: Erste Erfahrungen mit Telepräsenzrobotik in der häuslichen Schlaganfallversorgung

**DOI:** 10.1007/s00103-026-04295-7

**Published:** 2026-09-16

**Authors:** Sonja Haug, Karsten Weber, Edda Currle

**Affiliations:** Institut für Sozialforschung und Technikfolgenabschätzung, Ostbayerische Technische Hochschule (OTH) Regensburg, Postfach 12 03 27, 93025 Regensburg, Deutschland

**Keywords:** Rehabilitation, Selbstständigkeit, Mensch-Technik-Interaktion, Technologieakzeptanz, Ambulante Versorgung, Rehabilitation, Independence, Human-technology interaction, Technology acceptance, Outpatient care

## Abstract

**Hintergrund:**

Digitale Assistenzsysteme können Menschen mit Unterstützungsbedarf im Alltag begleiten, indem sie Informationen bereitstellen, Therapien unterstützen oder Kommunikation und soziale Teilhabe ermöglichen. Telepräsenzrobotik (TPR) umfasst physische, mobil im häuslichen Umfeld einsetzbare Systeme, die in der Versorgungspraxis bislang kaum etabliert sind. Ziel der Studie war es, Erwartungen, Erfahrungen und Akzeptanz von Schlaganfallpatient*innen, Angehörigen sowie Pflege- und Therapiepersonal gegenüber TPR zu untersuchen.

**Methoden:**

Vor (t1) und nach (t2) einer 24-wöchigen Testphase mit 2 TPR-Geräten (Feldphase 2021–2023) wurden Patient*innen und Angehörige standardisiert befragt (t1: 48 bzw. 53; t2: 39 bzw. 50). An einer Querschnittbefragung nahmen 31 Fachpersonen teil. Die Auswertung erfolgte mittels deskriptiver und bivariater Statistik. Ergänzend wurden persönliche Gespräche mit Patient*innen und Angehörigen mittels strukturierender Inhaltsanalyse nach Mayring ausgewertet.

**Ergebnisse:**

Die anfänglich hohen Erwartungen an TPR hinsichtlich Selbstständigkeit, Sicherheit, Kommunikation und Entlastung der Angehörigen im Alltag konnten häufig nicht erfüllt werden. Keines der Systeme wurde als überzeugender Alltagshelfer wahrgenommen. Autonome Mobilität und Sprachassistenz erwiesen sich als eingeschränkt hilfreich. Die einfache und flexible Tablet-Lösung wurde gegenüber TPR präferiert.

**Diskussion:**

TPR kann grundsätzlich die ambulante Versorgung unterstützen, ist jedoch aktuell noch nicht ausreichend an die Bedürfnisse der Patient*innen und häuslichen Bedingungen angepasst. Technische Einschränkungen, die Abhängigkeit von Angehörigen und die höhere Akzeptanz einfacherer Lösungen zeigen den Weiterentwicklungsbedarf für eine breitere Implementierung assistiver Systeme.

## Abstract (en)

**Background:**

Digital assistive systems can support everyday functioning of people needing help by providing information, supporting therapeutic processes and enabling communication and social participation. Telepresence robotics (TPR) comprises physically embodied, mobile systems that can be used in domestic environments. So far, the technology has not yet been widely implemented in healthcare practice. This study aimed to examine the expectations, experiences and acceptance of TPR among individuals with stroke, their relatives and healthcare staff.

**Methods:**

Before (t1) and after (t2) a 24-week trial phase involving two TPR devices (field phase 2021–2023), patients and relatives were surveyed using a standardised questionnaire (t1: 48 and 53 respectively; t2: 39 and 50). A cross-sectional survey involved 31 healthcare professionals. The data were analysed using descriptive and bivariate statistics. Additionally, personal interviews with patients and relatives were analysed using structuring qualitative content analysis according to Mayring.

**Results:**

The initially high expectations associated with TPR regarding independence, safety, communication and relief for relatives in everyday life were often not met. None of the systems were considered a convincing aid for everyday life. Autonomous mobility and voice assistance proved to be of limited help. The simple and flexible tablet solution was preferred over TPR.

**Discussion:**

While telepresence robotics has the potential to support outpatient care, it is not yet sufficiently adapted to patients’ needs and home environments. Technical limitations, dependence on family members and the higher acceptance of simpler technologies highlight the need for further development for a widespread implementation of assistive systems.

## Hintergrund

Die Entwicklung und Nutzung digitaler Assistenzsysteme in Pflege und Gesundheitsversorgung wird seit einigen Jahren zur Lösung der Herausforderungen des demografischen Wandels [1] und der damit verbundenen Folgen für eine alternde Gesellschaft [2–4] forciert. Eine besondere Versorgungsrelevanz kommt in diesem Kontext dem Schlaganfall zu, der aufgrund seiner hohen Prävalenz sowie seiner gesundheitlichen und sozialen Folgen eine erhebliche gesellschaftliche Bedeutung besitzt [5, 6]. Nach Abschluss der Akutversorgung und Rehabilitation stellt die nahtlose ambulante Anschlussversorgung für viele Betroffene eine besondere Herausforderung dar. Gleichzeitig gehen Schlaganfälle häufig mit Schwierigkeiten bei Alltagsbewältigung und sozialer Teilhabe einher [7]. In diesem Zusammenhang werden digitale Assistenzsysteme als mögliche Ergänzung bestehender Versorgungsangebote diskutiert, um die ambulante Nachsorge und die Bewältigung des Alltags von Schlaganfallbetroffenen zu unterstützen. Digitale Assistenzsysteme für ein gesundes und unabhängiges Leben sollen zum einen von Betroffenen selbst genutzt werden und zum anderen Angehörige, Gesundheits- und Pflegepersonal unterstützen.

Trotz langjähriger, erheblicher Investitionen in Forschung und Entwicklung digitaler Assistenztechnologien hat dies bislang nicht zu einer sichtbaren Verbreitung in der Versorgungspraxis geführt [8]. Insbesondere für die ambulante Versorgung von Schlaganfallbetroffenen liegen bislang nur wenige Erkenntnisse vor. Vor diesem Hintergrund wurde die Pilotstudie „DeinHaus4.0 Oberpfalz – TePUS (Telepräsenzroboter für die Pflege und Unterstützung von Schlaganfallpatientinnen und -patienten)“ zum ambulanten Einsatz digitaler Assistenzsysteme nach einem Schlaganfall durchgeführt.

Im Mittelpunkt der Studie stand der Einsatz von Telepräsenzrobotik (TPR) im häuslichen Umfeld. Telepräsenzroboter sind physische, mobil im häuslichen Umfeld einsetzbare Systeme, die sich innerhalb einer festgelegten Umgebung bewegen. Sie werden (über Sprache) gesteuert und ermöglichen die Kommunikation über Distanz [9]. In der Studie wurde TPR für ergänzende therapeutische Interventionen (Teletherapie und Telenursing) sowie für appbasierte Therapie eingesetzt. Ziel der Studie war die Evaluation der Umsetzbarkeit, Wirkung, Risiken und Akzeptanz einer TPR-gestützten Ergänzung der ambulanten Versorgung nach einem Schlaganfall. Untersucht wurde insbesondere, inwieweit TPR zur Verbesserung der Anschlussversorgung und zur Unterstützung eines selbstständigen Verbleibs von Schlaganfallbetroffenen im eigenen Zuhause beitragen kann. Die ergänzende Versorgung zielte aus diesem Grund über die Wiederherstellung motorischer und sprachlicher Funktionen hinaus darauf ab, einen Beitrag zur Aufrechterhaltung eines selbstständigen Lebens im eigenen Zuhause zu leisten sowie den Erwerb von Kompetenzen für eine erfolgreiche Anpassung an schlaganfallbedingte Einschränkungen zu unterstützen [10]. Die häufig berichteten besonderen Herausforderungen aufgrund funktioneller Bewegungseinschränkungen sollten über die Einbeziehung eines autonom beweglichen, sprachgesteuerten Systems in die Analyse adressiert werden.

Mit der Erprobung digitaler Systeme unter Realbedingungen schließt die interdisziplinäre Pilotstudie eine Lücke in der evidenzbasierten Pflege- und Therapieforschung. Die Ergebnisse zu den ergänzenden Pflege‑, Physiotherapie- und Logopädie-Anwendungen wurden bereits an anderer Stelle veröffentlicht [11] und sind nicht Gegenstand dieses Beitrags.

Der vorliegende Beitrag ist im Rahmen der sozialwissenschaftlichen Begleitstudie entstanden, die als eigenständige Studie zur genannten Pilotstudie konzipiert ist. Er geht darauf ein, wie Patient*innen nach einem Schlaganfall, deren Angehörige sowie Pflege- und Therapiefachkräfte den Einsatz von TPR im häuslichen Versorgungssetting bewerten. Im Mittelpunkt steht die Frage, welche Bedeutung die Technologie aus Sicht der beteiligten Akteursgruppen für die Alltagsbewältigung hat und wie sich die Einschätzungen vor und nach der Nutzung der Technologie verändern. Dabei werden die für eine selbstständige Alltagsbewältigung relevanten *sozialen Aspekte* der Förderung der Selbstständigkeit, Sicherheit, sozialen Teilhabe, Kommunikation sowie die mögliche Entlastung von Angehörigen betrachtet. Für diese Aspekte wird analysiert, welche Erwartungen die Beteiligten vor der Nutzung mit den Systemen verbinden und inwieweit sich diese Erwartungen nach Abschluss der Feldphase bestätigen oder verändern.

Ein weiterer Schwerpunkt liegt auf der Bewertung *zentraler technischer Eigenschaften* der eingesetzten Systeme. Untersucht wird, wie die Befragten die Alltagstauglichkeit der TPR sowie spezifische Funktionen wie Sprachsteuerung und autonome Beweglichkeit beurteilen. Übergeordnetes Ziel der sozialwissenschaftlichen Begleitstudie ist die Untersuchung der Nutzungsakzeptanz von TPR. Diese wird für die einzelnen Gruppen abschließend dargestellt und es wird darauf eingegangen, wie das tatsächliche Nutzungsverhalten im Vergleich der eingesetzten Systeme ausfiel.

Während frühere Publikationen zur Begleitstudie das zugrunde liegende Technikakzeptanzmodell (TA-Modell), erste Befunde [12–14] sowie einzelne Akteursgruppen [15, 16] thematisierten, führt der vorliegende Beitrag die verschiedenen Perspektiven von Patient*innen, Angehörigen und Pflege- und Therapiefachkräften systematisch zusammen und wertet sie vergleichend aus. Ziel ist es, ein differenziertes Bild durch neue Erkenntnisse zu zeichnen, welche Potenziale und Grenzen TPR für die Unterstützung des selbstständigen Lebens von Schlaganfallbetroffenen im häuslichen Umfeld aufweist.

## Methoden

### Studiendesign und Stichprobe

**Abb. 1:**
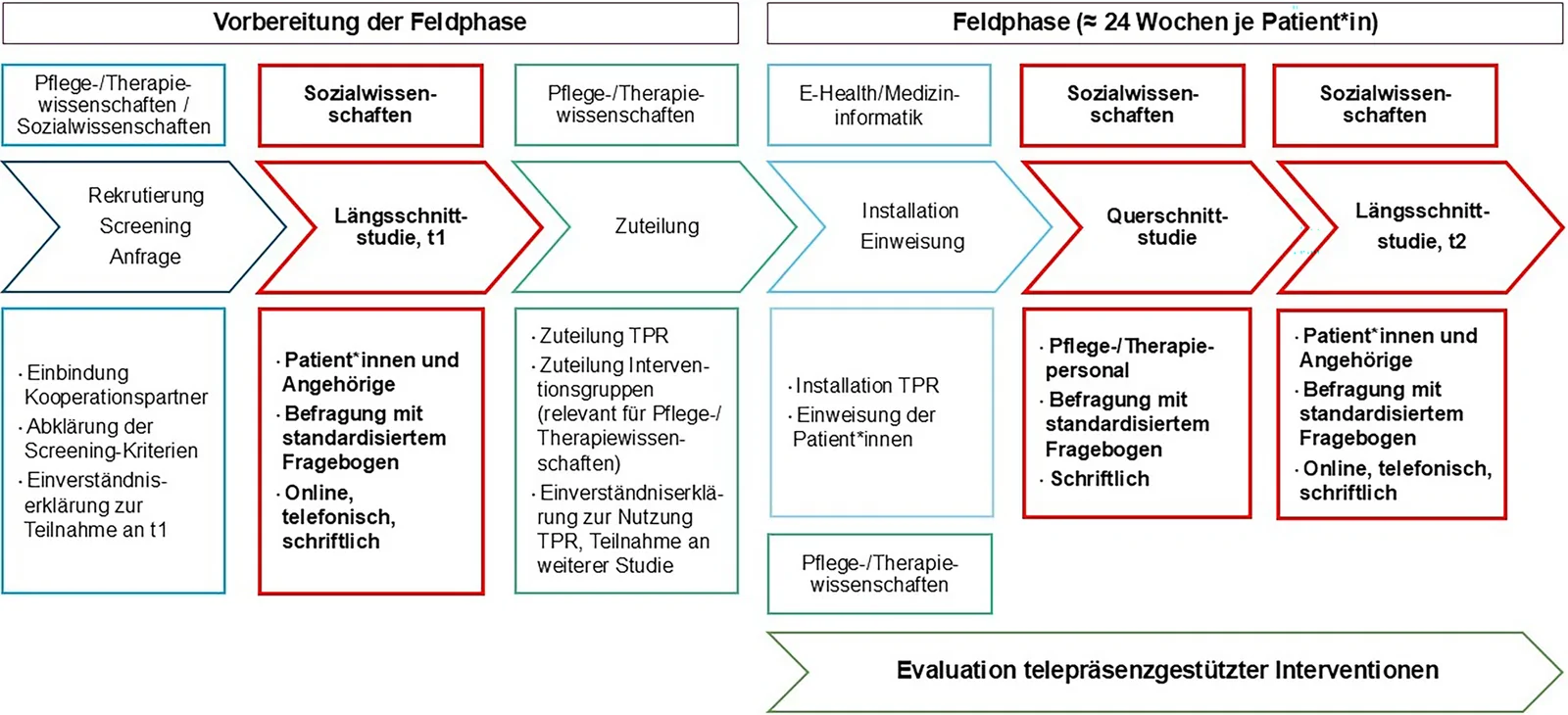
Einbettung der standardisierten Befragungen der sozialwissenschaftlichen Begleitstudie in die Feldphase der Pilotstudie. *TPR* Telepräsenzrobotik

Die sozialwissenschaftliche Begleitstudie war in den Ablauf der Feldphase der Pilotstudie integriert (Abb. 1). Die Perspektiven von Patient*innen und Angehörigen wurden in einem Prätest-Posttest-Design vor und nach Abschluss der Feldphase erhoben. Das Längsschnittdesign, das Einstellungs- und Verhaltensakzeptanz (Erklärung s. nächster Abschnitt) sowie die dafür relevanten Faktoren zu 2 Zeitpunkten erfasst, erlaubt den Vergleich der Erwartungen vor Beginn der Feldphase mit den Erfahrungen nach deren Abschluss. Ergänzend wurden die Einstellungen des im häuslichen Umfeld der Teilnehmenden tätigen Pflege- und Therapiepersonals in einer Querschnittbefragung während der Testphase erhoben [17].

**Abb. 2:**
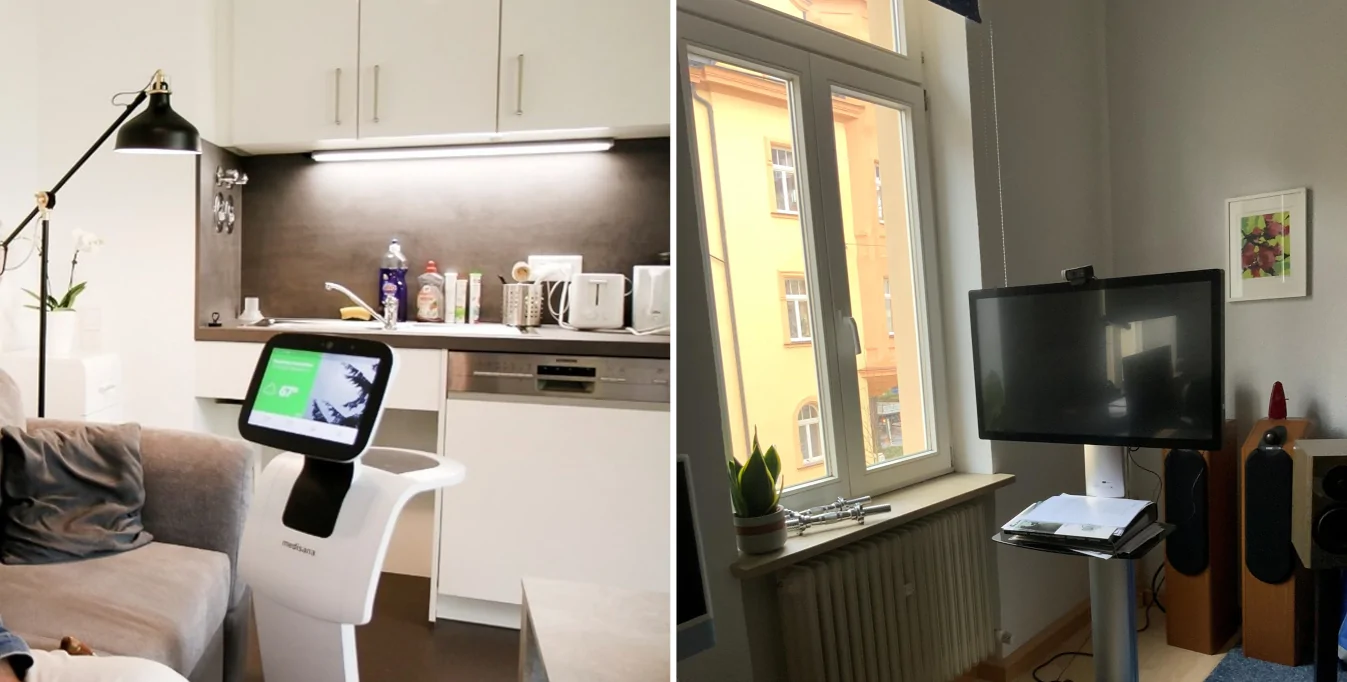
In der Pilotstudie getestete Telepräsenzrobotik im häuslichen Umfeld. *Links*: autonom beweglicher Telepräsenzroboter (temi), © Marco Linke, Manntau; *rechts*: statischer Telepräsenzroboter (DIY), © Edda Currle, OTH Regensburg. Beide Geräte wurden durch ein zusätzliches Tablet ergänzt.

Die beiden in der Feldphase der Pilotstudie eingesetzten TPR-Systeme (Abb. 2) verfügten über eine audiovisuelle Kommunikationsschnittstelle für Videokommunikation, Teletherapie und Telenursing, die die zentrale Funktionalität des Nutzungsszenarios darstellte. Neben dem kommerziell vertriebenen TPR temi©(temi Global Ltd., New York, NY, USA; Vertrieb als „medisana temi“ durch medisana GmbH, Neuss, Deutschland), der über autonome Navigationsmöglichkeiten und Sprachsteuerung verfügte, kam ein statisches Do-it-yourself-System (DIY) zum Einsatz, das für die Studie aus frei verfügbaren Komponenten entwickelt wurde und einen größeren Bildschirm aufwies. Den Teilnehmenden wurde fortlaufend und alternierend eines der beiden Systeme für die gesamte Testphase zugewiesen, sodass beide Systeme unter vergleichbaren Bedingungen erprobt werden konnten. Ein zusätzlich über den gesamten Untersuchungszeitraum zur Verfügung gestelltes Tablet erlaubte allen Teilnehmenden eine vom jeweiligen TPR unabhängige Nutzung [18].

Die therapeutischen Interventionen umfassten psychosoziale Unterstützung, logopädische und physiotherapeutische Anwendungen, die teils synchron in Live-Sitzungen, teils asynchron über appbasierte Eigenübungen durchgeführt wurden [19]. Die durchschnittliche Nutzungsdauer der TPR betrug 24 Wochen während der Feldphase (2021–2023). Die Stichprobe der Prätest-Befragungen t1 umfasste 48 Patient*innen und 53 Angehörige. 39 Patient*innen und 50 Angehörige nahmen an den Posttest-Befragungen t2 teil. Die Stichprobe der Querschnittstudie des Pflege- und Therapiepersonals umfasste 31 Personen.

### Theoretische Grundlage und Erhebungsinstrument

Die Technologieakzeptanzforschung untersucht auf Grundlage geeigneter theoretischer Modelle Einflussfaktoren auf den Prozess, der bei einem Individuum zur Nutzung oder Nichtnutzung einer Technologie führt. Einstellungs-Verhaltens-Modelle wie die „Theory of Planned Behavior“ [20] gehen davon aus, dass die Einstellungsakzeptanz („Einstellung zur Nutzung“) einen positiven Einfluss auf die Verhaltensakzeptanz („Nutzungsabsicht/-bereitschaft“) hat. Grundlage der Datenerhebung der Begleitstudie bildet ein an den Forschungskontext angepasstes Technologieakzeptanzmodell nach Davis [21], das bereits in früheren Arbeiten beschrieben wurde.

Die theoretischen Konstrukte des Modells, d. h. die zu untersuchenden Merkmale, wie die wahrgenommene Nützlichkeit oder die Einstellung zur Nutzung, wurden durch geeignete Messgrößen (Operationalisierung) abgebildet und ihre Ausprägungen mittels vollstandardisierter, für die Akteursgruppen weitgehend identischer Fragebögen erhoben. Diese basierten teilweise auf etablierten Messinstrumenten und wurden durch an den Nutzungskontext angepasste Fragen ergänzt [12]. Die Operationalisierung der für die vorliegende Arbeit relevanten Konstrukte zur Analyse der Erwartungen und Erfahrungen bezüglich der wahrgenommenen Nützlichkeit der TPR, ihrer Unterstützung bei der Alltagsbewältigung sowie der relevanten sozialen Aspekte ist in Tab. 1 dargestellt. Die Einstellung zur Nutzung bildet die zentrale abhängige Zielvariable des Modells und steht im Mittelpunkt der Analyse der Akzeptanz der TPR. Angehörige und Fachpersonal wurden zu ihren Einstellungen zur Nutzung von TPR bei der Betreuung der Patient*innen befragt. Auf der Grundlage des TA-Modells wird davon ausgegangen, dass soziale Aspekte sowie das Konstrukt „Wahrgenommene Nützlichkeit“ die Einstellung zur Nutzung positiv beeinflussen.

**Tab. 1:** Operationalisierung der für die Alltagsbewältigung relevanten Konstrukte in den Fragebögen der standardisierten Befragungen (t1 und t2 Patient*innen und Angehörige; Querschnittbefragung des Pflege- und Therapiepersonals)

Konstrukte	Operationalisierung (Items)
*Soziale Aspekte der Unterstützung bei der Alltagsbewältigung*	
Förderung der Selbstständigkeit	„Der TPR trägt dazu bei, dass ich als Schlaganfallpatient länger zu Hause leben kann“
Sicherheit im Alltag	„Mit dem TPR zu Hause fühle ich mich sicher“
Soziale Teilhabe	„Mein persönlicher Kontakt zu anderen Menschen kommt beim Einsatz des TPR zu kurz“
Kommunikation	„Der TPR erleichtert die Kommunikation mit Angehörigen und Fremden“
Entlastung von Angehörigen	„Der TPR trägt zur Entlastung von Angehörigen bei“
*Wahrgenommene Nützlichkeit zentraler technischer Eigenschaften*	
Alltagstauglichkeit	„Der TPR macht meinen Alltag zu Haus alles in allem leichter“
Flexibilität	„Ein Telepräsenzroboter muss flexibel an verschiedene Situationen oder Krankheitsstadien von mir anpassbar sein“
Zuverlässigkeit	„Ein Telepräsenzroboter muss zuverlässig funktionieren“
Wartungsfreundlichkeit	„Kundendienst, Wartung und Service eines Telepräsenzroboters müssen unkompliziert sein“
Autonome Beweglichkeit	„Die Möglichkeit, den TPR an verschiedenen Stellen in der Wohnung/im Haus zu nutzen, finde ich …“
Sprachsteuerung	„Die Möglichkeit, eine Sprachsteuerung zu nutzen, finde ich …“
*Einstellungsakzeptanz (Einstellung zur Nutzung)*	
Patient*innen	t1: „Einmal angenommen, Sie können ein Gerät nutzen. Können Sie sich das vorstellen?“ t2: „… auch zukünftig nutzen. Können Sie sich das vorstellen?“
Angehörige/Fachpersonal	„Einmal angenommen, eines dieser Geräte würde Ihrer/Ihrem Angehörigen resp. Patient*in dauerhaft zur Verfügung gestellt. Können Sie sich das vorstellen?“

### Datenerhebung und Datenanalyse

Die Befragungen der Längsschnittstudie wurden über standardisierte Fragebögen mit Patient*innen und Angehörigen vor Ort, telefonisch, schriftlich oder über Videokommunikation durchgeführt. Die Querschnittbefragung des Fachpersonals erfolgte über schriftliche Fragebögen. Die Auswertung der standardisierten Daten mit IBM SPSS 29 umfasst deskriptive und bivariate schließende Statistik zur Untersuchung von Gruppenunterschieden und Veränderungen [22]. Mittelwertvergleiche der für die Alltagsbewältigung relevanten sozialen Aspekte wurden genutzt, um zu beurteilen, ob die auf einer 5‑stufigen Likert-Skala erhobenen Bewertungen (1 stimme gar nicht zu, 5 stimme völlig zu) eher positiv ausfielen (Werte > 3) oder auf bestehende Nutzungsbarrieren (Werte < 3) verweisen. Mithilfe von t‑Tests wurde geprüft, ob sich die Mittelwerte zwischen den befragten Gruppen statistisch signifikant voneinander unterscheiden. Ergänzend wurden die bei persönlichen Gesprächen erhobenen Aussagen von Patient*innen und Angehörigen qualitativ ausgewertet. Die qualitativen Daten wurden mit strukturierender Inhaltsanalyse nach Mayring auf der Grundlage eines deduktiv aus dem TA-Modell und den Fragebogenkonstrukten entwickelten Kategoriensystems kodiert und mit MAXQDA qualitativ inhaltsanalytisch ausgewertet [23].

## Ergebnisse

### Beschreibung der Stichproben

An der Prätest-Befragung t1 nahmen insgesamt 48 Patient*innen teil (Durchschnittsalter 62,4, max. = 86, min. = 34, SD = 10,9; 71 % männlich, 29 % weiblich), 39 Patient*innen an der Posttest-Befragung t2. Die 53 Angehörigen waren überwiegend weiblich und im Durchschnitt 53 Jahre alt. An der Posttest-Befragung beteiligten sich 50 Angehörige. Den Fragebogen für das Pflege- und Therapiepersonal beantworteten 31 Personen. Die Teilnehmenden waren im Durchschnitt 41 Jahre alt, mit einem Frauenanteil von knapp 84 % war auch diese Stichprobe überwiegend weiblich.

### Soziale Aspekte der Unterstützung bei der Alltagsbewältigung: Erwartungen und Erfahrungen

#### Förderung der Selbstständigkeit.

Die Analyse der ersten standardisierten Befragung zeigt, dass insbesondere die Patient*innen vor Beginn der Testphase erwarteten, durch die Nutzung der Systeme ihre Selbstständigkeit zu stärken und dadurch länger zu Hause leben zu können (Tab. 2). Der von 3,81 auf 2,81 gesunkene Mittelwert (MW) drückt aus, dass die Bewertung zur Förderung ihrer Selbständigkeit nach Abschluss der Nutzungsphase deutlich negativer ausfiel als die zuvor geäußerten Erwartungen. Im Gegensatz dazu verbanden die Angehörigen weniger Hoffnung damit, die TPR stärke die Selbstständigkeit der Betroffenen (MW: 3,53). Auch ihre Bewertung fiel nach der Testphase negativer aus als zuvor (MW: 3,10). Das während der Testphase befragte Fachpersonal hingegen bewertete den Aspekt der Förderung der Selbstständigkeit mit durchschnittlich 2,96 weitaus weniger positiv.

**Tab. 2:** Bewertung ausgewählter sozialer Aspekte der Unterstützung bei der Alltagsbewältigung durch Patient*innen (PA), Angehörige (AN) vor und nach der Testphase (Zeitpunkte t1 und t2) und durch Pflege- und Therapiefachpersonal (PT) in der Querschnittbefragung während der Testphase sowie Mittelwertvergleiche der Aspekte bei den befragten Gruppen

	Bewertung der sozialen Aspekte			Mittelwertvergleiche der befragten Gruppen		
Prätest-Befragung von Patient*innen und Angehörigen (t1) und Querschnittbefragung Fachpersonal						
–	Patient*innen *t1*	*Angehörige* *t1*	*Pflege- und Therapiefachpersonal* *Querschnittbefragung*	*PA-AN*	*PA-PT*	*AN-PT*
Selbstständigkeit	3,81	3,53	2,96	n. s.	**	*
Sicherheit	3,42	2,90	3,04	n. s.	n. s.	n. s.
Soziale Teilhabe	4,26	3,98	3,00	n. s.	**	**
Kommunikation	3,50	2,93	3,75	**	n. s.	**
Entlastung von Angehörigen	3,66	3,09	3,55	**	n. s.	*
Posttest-Befragungvon Patient*innen und Angehörigen (t2) und Querschnittbefragung Fachpersonal						
*–*	Patient*innen *t2*	*Angehörige* *t2*	*Pflege- und Therapiefachpersonal* *Querschnittbefragung*	*PA-AN*	*PA-PT*	*AN-PT*
Selbstständigkeit	2,81	3,10	2,96	n. s.	n. s.	n. s.
Sicherheit	2,94	2,94	3,04	n. s.	n. s.	n. s.
Soziale Teilhabe	4,06	4,06	3,00	n. s.	n. s.	n. s.
Kommunikation	2,56	3,08	3,75	n. s.	n. s.	**
Entlastung von Angehörigen	2,76	3,57	3,55	n. s.	**	n. s.

Skalenwert 1: stimme gar nicht zu, 5: stimme völlig zu; Mittelwerte unter 3 entsprechen einer eher negativen Bewertung, Werte über 3 einer positiven Bewertung

t‑Test *p ≤ 0,05, **p ≤ 0,01. *n.s.* nicht signifikant

#### Sicherheit im Alltag.

Während Angehörige den Beitrag der TPR zu einem höheren Sicherheitsgefühl im Alltag bereits zu Beginn eher negativ einschätzten (MW: 2,90), fiel die Bewertung durch Patient*innen (MW: 3,42) zunächst moderat aus. Dies gilt ebenfalls für die Fachkräfte (MW: 3,04). Im Verlauf der Nutzung wurde jedoch auch bei den Patient*innen die Bewertung schlechter (MW: 2,94). Am Ende der Feldphase wurden die Assistenzsysteme von keiner Akteursgruppe als relevanter Beitrag zur Erhöhung der Sicherheit im häuslichen Umfeld wahrgenommen.

#### Soziale Teilhabe und Kommunikation.

Vor Beginn der Nutzung erwarteten die meisten befragten Patient*innen keine Beeinträchtigung ihrer sozialen Teilhabe (MW: 4,26). Gleichzeitig hofften sie auf verbesserte Kommunikationsmöglichkeiten außerhalb des Haushalts und drückten ihre Erwartungen mit einem Mittelwert von 3,50 aus. Diese erfüllten sich nur teilweise. Insbesondere die Kommunikation nach außen wurde nach der Nutzungsphase nicht mehr positiv bewertet (MW: 2,56). Die Angehörigen äußerten in keiner der Befragungen die Befürchtung, die soziale Teilhabe werde durch eine Nutzung von TPR eingeschränkt (MW: 3,98 bzw. 4,06), und standen einer Verbesserung der Kommunikationsmöglichkeiten weitgehend neutral gegenüber (MW: 2,93 bzw. 3,08). Das befragte Pflege- und Therapiepersonal bewertete mögliche Auswirkungen auf den persönlichen Kontakt zu anderen Menschen bei einem Mittelwert von 3,00 kritischer als die übrigen Gruppen, sahen die verbesserten Kommunikationsmöglichkeiten hingegen positiver (MW: 3,75).

#### Entlastung von Angehörigen.

Die mögliche Entlastung von Angehörigen wurde von allen 3 Akteursgruppen bereits vor Beginn der Feldphase als potenzieller Vorteil angesehen. Am skeptischsten äußerten sich die Angehörigen (MW: 3,09 gegenüber 3,66 bei Patient*innen und 3,55 beim Fachpersonal), mit signifikantem Unterschied zu den beiden anderen Gruppen. Nach der Nutzungsphase zeigte sich eine differenzierte Entwicklung: Angehörige nahmen die TPR stärker als Unterstützung wahr als noch zu Beginn der Studie und bewerteten die Entlastungsfunktion nun positiver (MW: 3,57). Die Patient*innen äußerten sich zunächst positiver als ihre Angehörigen, beurteilten nach der Nutzung den Beitrag der Systeme zur Entlastung ihrer Angehörigen jedoch negativer als zunächst erwartet (MW: 2,76). Insgesamt deuten die Ergebnisse darauf hin, dass die Wirkung von den Angehörigen selbst stärker wahrgenommen wurde als von den Patient*innen.

### Bewertung zentraler technischer Eigenschaften

#### Alltagstauglichkeit.

Die vor Nutzungsbeginn durchgeführte erste Befragung zu den Erwartungen der Patient*innen im Hinblick auf die Nützlichkeit der Systeme verdeutlichte, dass diese insbesondere Zuverlässigkeit, Wartungsfreundlichkeit und Flexibilität als wichtig oder sehr wichtig erachteten, während dem Design wenig Bedeutung zugemessen wurde. Auch Datenschutzaspekte spielten in der Prätest-Befragung eine vergleichsweise untergeordnete Rolle ([24]; zur Operationalisierung: Tab. 1). Die Erfahrungen während der Feldphase zeigen, dass zentrale Erwartungen an die Nützlichkeit der Systeme nicht erfüllt werden konnten. Aus Sicht der Patient*innen erreichte das autonom bewegliche System in der zweiten Befragung in den Kategorien „Alltagstauglichkeit“ und „Flexibilität“ zwar leicht bessere Bewertungen als das statische System. Bei der von den Patient*innen in der ersten Befragung als besonders wichtig eingeschätzten Zuverlässigkeit schnitt das statische System jedoch besser ab. Keines der beiden Systeme wurde jedoch nach der Testphase als wirklich nützlicher Alltagshelfer bewertet: Weniger als ein Viertel der Befragten stimmte der Aussage zu, das verwendete System mache ihren Alltag alles in allem leichter. Vor Beginn der Testphase hatten noch rund 70 % der Teilnehmenden positive Erwartungen in Bezug auf diesen Aspekt geäußert. Vor allem der temi© hat in diesem Punkt die Vorstellungen enttäuscht [15]: Neben technischen Problemen, Unzuverlässigkeit oder fehlender stabiler Netzverbindungen wurden in den persönlichen Gesprächen vor allem Probleme mit der Sprachsteuerung und der autonomen Mobilität angeführt.

#### Autonome Beweglichkeit.

Die Evaluation der über den Sprachassistenten steuerbaren autonomen Beweglichkeit bezieht sich auf den temi©, da der DIY nicht autonom beweglich war (Abb. 2). Bereits in einer frühen Testphase zeigten sich im Vergleich zu Laborbedingungen erhebliche Schwierigkeiten bei seinem Einsatz im häuslichen Umfeld [18]. Im Praxiseinsatz erwiesen sich Teppiche, Türschwellen, Möbel und Treppen als wesentliche Hindernisse, sodass der mobile TPR auf nur einer Etage nutzbar war:

„Die Probleme sind schon damit losgegangen, dass er nicht über den Teppich kommt und über die Türschwelle“ (P22).

„Den temi kann ich nicht so einfach ins andere Zimmer mitnehmen. Über die Schwelle kommt er nicht drüber, die ist 2 cm hoch. Beim Teppich schaukelt er schon sehr, der ist 1 cm“ (G24).

„Er hat nicht funktioniert wegen der Stolperfallen. Neun von zehn Mal findet er vom Tisch nicht in die Basisstation. Er hat definitiv Orientierungsprobleme“ (P22).

**Abb. 3:**
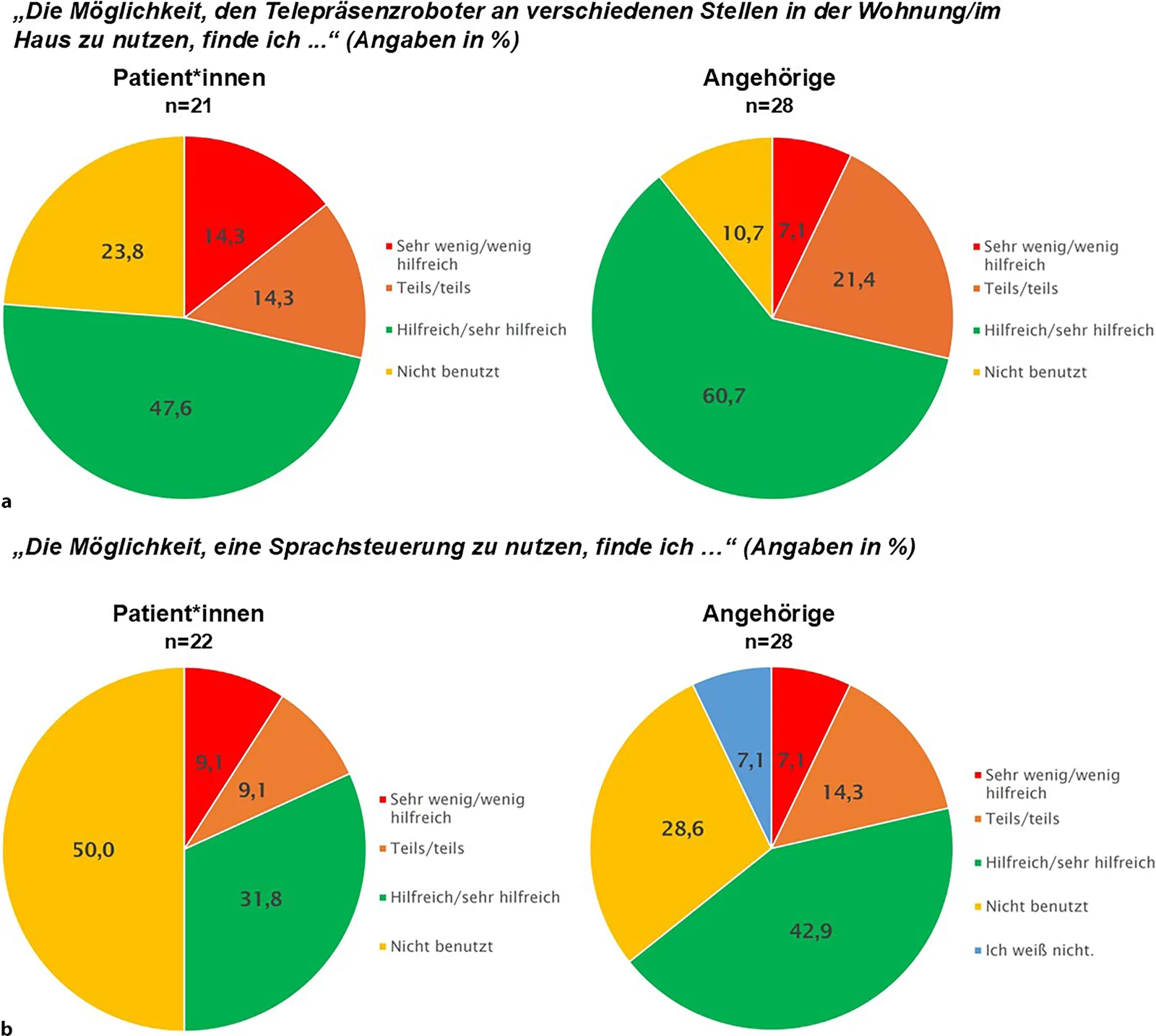
Wahrgenommene Nützlichkeit laut Posttest-Befragung von **a** autonomer Beweglichkeit und **b** Sprachsteuerung des Telepräsenzroboters durch Patienten*innen und Angehörige nach der Testphase (Zeitpunkt t2)

Insgesamt konnte nur knapp die Hälfte der Patient*innen in dieser Funktion einen Nutzen erkennen. Etwa ein Viertel nutzte sie gar nicht. Angehörige bewerteten die autonomen Bewegungsmöglichkeiten insgesamt positiver (Abb. 3a).

#### Sprachsteuerung.

Auch die Sprachsteuerung des temi© wurde von den Patient*innen differenziert bewertet. Insbesondere für Personen mit Sprach- oder Sprechbeeinträchtigungen erwies sie sich als nur eingeschränkt nutzbar. Etwa die Hälfte der Proband*innen verzichtete auf ihren Gebrauch:

„Die Sprachsteuerung habe ich nicht benutzt, um ihn zu bewegen, er hätte es eh nicht über den Teppich geschafft. Ich habe ihn drüber getragen“ (G15).

Ein Drittel der tatsächlichen Nutzer*innen bewerteten die Sprachsteuerung als (sehr) hilfreich; Angehörige äußerten sich positiver (Abb. 3b). Darüber hinaus gab nur etwas mehr als ein Viertel der Patient*innen an, gut oder sehr gut mit der Sprachsteuerung zurechtgekommen zu sein, während die übrigen Personen die Funktion nur eingeschränkt oder sogar schlecht bedienen konnten.

### Einstellungen zur Nutzung als zentrale Zielvariable und Akzeptanz der eingesetzten Technik im Vergleich

**Abb. 4:**
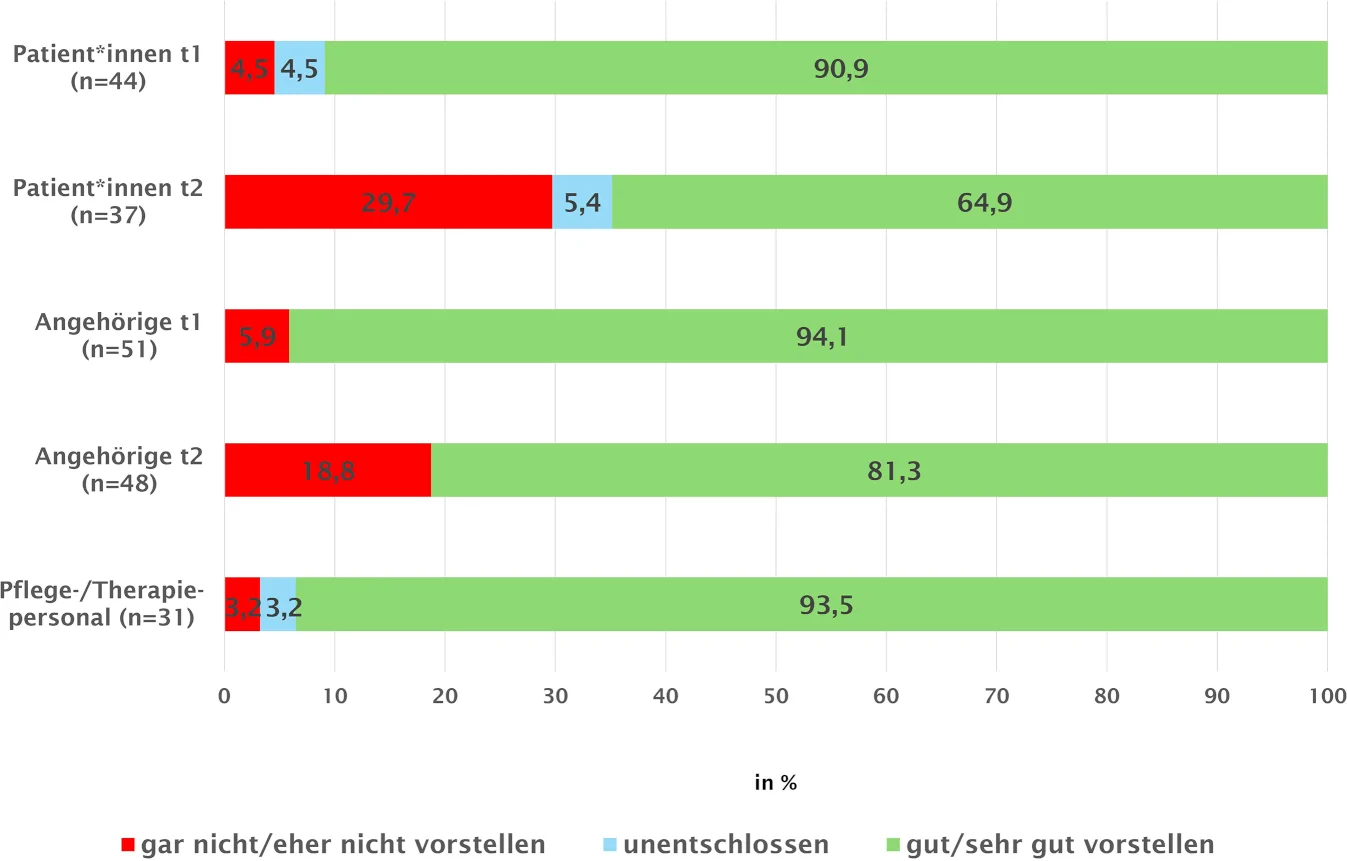
Einstellungen zur Nutzung der Telepräsenzrobotik bei Patient*innen und Angehörigen in der Prätest- (t1) und Posttest-Befragung (t2) sowie beim Pflege- und Therapiepersonal in der Querschnittbefragung

Die zu Beginn der Feldphase erwartungsgemäß hohe Einstellungsakzeptanz verringerte sich im Verlauf der Nutzung, blieb aber bei Patient*innen und Angehörigen mehrheitlich positiv. Rund 2 Drittel der Patient*innen konnten es sich auch nach Abschluss der Testphase (sehr) gut vorstellen, TPR zukünftig zu nutzen. Bei den Angehörigen lag die Zustimmung weiterhin bei über 80 %. Die höchste Akzeptanz zeigte das in der Querschnittstudie befragte Pflege- und Therapiepersonal, das sich eine weitere Nutzung im Versorgungskontext am ehesten vorstellen konnte (Abb. 4).

Die Möglichkeit, das Tablet unabhängig vom robotischen System flexibel in unterschiedlichen Räumen zu nutzen, wurde nahezu durchgängig positiv beurteilt. Die Analyse des tatsächlichen Nutzungsverhaltens der eingesetzten Technik zeigt demzufolge, dass es im Versorgungsalltag deutlich häufiger verwendet wurde als die TPR: Mehr als 2 Drittel der Patient*innen nutzten überwiegend das Tablet, während nur knapp 18 % den TPR bevorzugten. Etwas mehr als 15 % setzten die Geräte in etwa gleich häufig ein. Die qualitativen Befunde legen ein typisches Nutzungsmuster nahe: Zeitlich asynchrone, eigenständig nutzbare Anwendungen aus dem Therapie- und Pflegebereich wurden bevorzugt vom Tablet aus gestartet, während der TPR zum Teil ausschließlich für die Teilnahme an Videosprechstunden aktiviert wurde. Die Testpersonen des DIY griffen dabei häufiger auf das Tablet zurück als die Testpersonen des temi© [15].

## Diskussion

Die Ergebnisse zeigen, dass die anfänglich überwiegend positiven Erwartungen an die TPR hinsichtlich der Unterstützung der Alltagsbewältigung im Verlauf der Nutzung nur teilweise bestätigt wurden. Insbesondere die von Patient*innen erhoffte Förderung von Selbstständigkeit, Sicherheit und Kommunikationsmöglichkeiten wurde nach der Testphase deutlich kritischer bewertet. Dagegen wurde eine mögliche Entlastung von Angehörigen von allen Akteursgruppen als positive Erfahrung wahrgenommen, wobei dieser Effekt insbesondere von den Angehörigen selbst bestätigt wurde. Die eingeschränkte Erfüllung der Erwartungen ist unter anderem auf technische und funktionale Herausforderungen zurückzuführen. Probleme bei der autonomen Navigation und der Sprachsteuerung beeinträchtigten die Alltagstauglichkeit der Systeme erheblich und führten dazu, dass die TPR deutlich seltener genutzt wurden als das ergänzende Tablet. Trotz dieser Einschränkungen blieb die grundsätzliche Akzeptanz der Technologie bei Patient*innen, Angehörigen und Fachpersonal überwiegend positiv, was auf ein grundsätzlich vorhandenes Potenzial der Technologie hinweist.

Die positiven Erwartungen an die in der Pilotstudie getesteten TPR in Bezug auf Selbstständigkeitsgewinn stehen im Einklang mit weiteren Studien, die einen positiven Effekt von Aufklärung, Beratung und Information sowie dem Training von Alltagsaktivitäten in Verbindung mit psychosozialer Unterstützung auf das Selbstmanagement von Schlaganfallpatient*innen beschreiben [25]. Die Ergebnisse der vorliegenden Studie zeigen jedoch, dass sich diese Erwartungen im Verlauf der Nutzung nur teilweise bestätigten. Das zunächst wahrgenommene Potenzial der Technologie für die Unterstützung der Selbstständigkeit führte nicht zur Wahrnehmung eines entsprechenden Nutzens im Alltag.

Die Befunde legen vielmehr nahe, dass die tatsächliche Versorgungswirkung von mehreren Bedingungen abhängt. Hierzu zählen insbesondere die technische Zuverlässigkeit der eingesetzten Systeme, die Passung der Technologie an die individuellen Bedarfe der Nutzenden sowie ihre Einbettung in den häuslichen Alltag. Erst wenn diese Voraussetzungen erfüllt sind, können die von den Teilnehmenden erwarteten Unterstützungsleistungen im Alltag für ein erfolgreiches Selbstmanagement wirksam werden. Die vorliegenden Ergebnisse bestätigen somit die in der Literatur hervorgehobene Bedeutung einer bedarfsorientierten und individualisierten Gestaltung digitaler Unterstützungsangebote [26].

Unabhängig von der Frage nach dem konkreten Nutzen für die Alltagsbewältigung erwies sich die Zuverlässigkeit der eingesetzten TPR als wichtiger Einflussfaktor auf die Bewertung der Systeme. Dies deckt sich mit Befunden weiterer Studien, die Zuverlässigkeit als zentralen Faktor für die Akzeptanz digitaler Technologien beschreiben [27]. Insgesamt deuten die Ergebnisse darauf hin, dass TPR grundsätzlich als sinnvolle Ergänzung ambulanter Pflege- und Therapieangebote wahrgenommen wurden. Die erhoffte Unterstützung bei der Bewältigung alltäglicher Herausforderungen wurde jedoch von Nutzenden nur eingeschränkt erlebt.

Die in der Literatur beschriebenen unterschiedlichen Bedürfnisse älterer Menschen oder Personen mit Mobilitätseinschränkungen [28] spiegeln sich in den vorliegenden Ergebnissen wider. Sie verdeutlichen, dass die derzeit am Markt erhältlichen TPR künftig stärker sowohl an die Bedürfnisse der Nutzenden als auch an die Umgebungsbedingungen in der häuslichen Versorgung angepasst werden müssen. Trotz der beschriebenen Einschränkungen fiel die Akzeptanz der getesteten Assistenzsysteme quer über die Akteursgruppen hinweg hoch aus. Gleichzeitig wurde das ergänzende Tablet als Gerät, das im Alltag vieler Menschen bereits angekommen ist, deutlich häufiger genutzt und positiver bewertet. Die Präferenz von Tablet vor Robotik lässt sich auch in anderen Studien beobachten [29]. Neu ist die daraus zu schließende Erkenntnis, dass der Bedarf an Pflege- und Therapieanwendungen im häuslichen Bereich mit einem handelsüblichen Tablet gedeckt werden könnte. Die zurückhaltende Zahlungsbereitschaft der Teilnehmenden verdeutlicht zudem, dass der wahrgenommene Mehrwert der TPR gegenüber Tablets begrenzt ist [15]. Datenschutzbedenken durch den Einsatz cloudbasierter Systeme im häuslichen Umfeld, wie sie in anderen Studien beschrieben werden [30], spielten in der vorliegenden Studie hingegen aufgrund der Einhaltung hoher Datenschutzanforderungen nur eine untergeordnete Rolle.

Die autonome Mobilität wurde unter den Bedingungen realer Wohnumgebungen als wenig praktikabel erfahren und beschrieben. Während frühere Untersuchungen diese Funktion für ältere Menschen im häuslichen Umfeld als essenziell beurteilten [28], zeigen die vorliegenden Ergebnisse, dass gerade diese Funktion an den Gegebenheiten häuslicher Umgebung scheitern kann. Diese Diskrepanz verdeutlicht die Notwendigkeit weiterer technischer Entwicklungen und Anpassungen. Ähnliche Befunde zeigen sich bei der Sprachsteuerung. Insbesondere für Personen mit schlaganfallbedingten Sprach- und Sprechstörungen erwies sie sich als nur bedingt geeignet und wurde wenig genutzt. Die Ergebnisse unterstreichen somit die Befunde früherer Studien zum Einsatz sprachbasierter Agenten [31, 32].

Zwar stellten Patient*innen der Benutzerfreundlichkeit beider TPR nach Abschluss der Testphase ein insgesamt positives Urteil aus. Einschränkend wurde jedoch berichtet, dass sie für die Nutzung von TPR auf die Hilfe von Angehörigen angewiesen waren. Dieser Befund wurde auch in anderen Studien beobachtet [32–34] und verweist auf die zentrale Rolle von Angehörigen bei der Einführung und der weiteren Nutzung digitaler Assistenzsysteme im ambulanten Kontext [35]. Die Ergebnisse zeigen, dass die für die Selbstständigkeit wirkende Barriere, ohne Angehörige das Haus nicht verlassen zu können, durch die Barriere ergänzt werden kann, ohne Angehörige das Assistenzsystem nicht bedienen zu können. Bestehende Abhängigkeiten und somit Einschränkungen der Selbstständigkeit werden somit nicht zwangsläufig reduziert, sondern durch weitere fortgeführt. Gleichzeitig gewinnt vor dem Hintergrund zunehmenden „Distance Caregiving“ [36] die Frage an Bedeutung, wie technische Unterstützung auch dann sichergestellt werden kann, wenn Angehörige nicht unmittelbar verfügbar sind. Die Rolle von Angehörigen im soziotechnischen Gefüge der Anwendung altersgerechter Assistenzsysteme bleibt also essenziell.

Die hohe Akzeptanz der befragten Pflege- und Therapiefachkräfte unterscheidet sich von Ergebnissen früherer Befragungen, in denen Pflegerobotik deutlich kritischer bewertet wurde [37]. Eine mögliche Erklärung liegt in den unterschiedlichen Anwendungsszenarien. Während in anderen Studien Robotik im Kontext direkter pflegerischer Tätigkeiten betrachtet wird, standen in der vorliegenden Studie Kommunikations- und Therapieunterstützung im Vordergrund. Darüber hinaus könnte die aktive Einbindung der Fachkräfte in die Erprobung zu einer realistischeren Einschätzung von Potenzialen und Grenzen beigetragen haben. Die vergleichsweise geringe Bedeutung von Datenschutzaspekten in der vorliegenden Untersuchung steht auf den ersten Blick im Widerspruch zu Bevölkerungsbefragungen, in denen dem Schutz von Gesundheitsdaten eine hohe Bedeutung zugemessen wird [38]. Die Ergebnisse legen jedoch nahe, dass im konkreten Versorgungskontext eine Abwägung zwischen Datenschutz und wahrgenommenem Nutzen vorgenommen wurde. Dies bedeutet jedoch nicht, dass Datenschutz vernachlässigt werden kann.

Die Ergebnisse sind vor dem Hintergrund mehrerer methodischer Limitationen zu interpretieren. Erstens handelt es sich um eine Studie mit einer kleinen Stichprobe, wodurch die statistische Aussagekraft begrenzt ist. Die Übertragbarkeit der Ergebnisse auf andere Zielgruppen oder Versorgungskontexte ist somit nur eingeschränkt möglich. Zudem kann nicht ausgeschlossen werden, dass sich insbesondere Personen mit einer grundsätzlich positiven Einstellung gegenüber Technik eher zur Studienteilnahme bereit erklärt haben. Als weitere Einschränkung ist zu berücksichtigen, dass die Bewertung der Systeme zu einem Zeitpunkt erfolgte (2021–2023), an dem die eingesetzten Technologien noch deutliche technische Limitationen aufwiesen. Die festgestellten Probleme bei Navigation oder Sprachsteuerung beeinflussten die Nutzungserfahrungen erheblich. Die Ergebnisse spiegeln daher das Potenzial von TPR zu einem Zeitpunkt wider, der den damaligen Entwicklungsstand der eingesetzten Systeme berücksichtigt.

Die wesentliche Stärke der Studie liegt in der Evaluation unter realen Bedingungen der häuslichen Versorgung, die im Gegensatz zu Untersuchungen in Labor- oder Simulationsumgebungen Erfahrungen aus dem tatsächlichen Versorgungsalltag abbilden. Zudem wurden mit Patient*innen, Angehörigen sowie Pflege- und Therapiefachkräften unterschiedliche Akteursgruppen einbezogen, wodurch eine umfassende Betrachtung von Akzeptanz der Systeme möglich wurde. Die Kombination quantitativer und qualitativer Daten erlaubt darüber hinaus, positive oder negative Bewertungen differenzierter nachzuvollziehen.

Die Ergebnisse verdeutlichen den Bedarf an weiterer Forschung zur alltagsnahen Implementierung von TPR im ambulanten Versorgungssetting. Zukünftige Forschung sollte sich daher nicht nur auf die technische Weiterentwicklung konzentrieren, sondern verstärkt untersuchen, unter welchen Bedingungen eingesetzte Systeme einen nachweisbaren Nutzen, und zwar für alle beteiligten Akteursgruppen, erzeugen. Insbesondere Fragen der langfristigen Nutzung, der Implementierung in bestehende Versorgungsstrukturen, der Rolle informeller Unterstützungssysteme sowie der gesundheitsökonomischen Tragfähigkeit erscheinen von zentraler Bedeutung. Zugleich gilt es zu klären, welche Patient*innengruppen besonders von TPR profitieren und wie autonome Navigation sowie Sprachsteuerung unter Alltagsbedingungen zuverlässig umgesetzt werden können. Die Ergebnisse zeigen, dass die Frage, welche technischen Funktionen von Assistenzsystemen aus Sicht der Nutzenden einen tatsächlichen Mehrwert gegenüber etablierten Technologien wie Tablets erzeugen, relevant ist. Zukünftige Studien sollten zudem mit größeren und heterogeneren Stichproben sowie längeren Beobachtungszeiträumen agieren.

Für die Versorgungspraxis zeigen die Ergebnisse, dass TPR gegenwärtig vor allem als ergänzendes Instrument und weniger als eigenständige Lösung für die Unterstützung der Alltagsbewältigung betrachtet werden sollte. Der größte praktische Nutzen scheint derzeit in der Unterstützung von Therapieanwendungen zu liegen. Für viele dieser Anwendungen erwiesen sich jedoch etablierte Technologien wie Tablets als ausreichend und teilweise sogar überlegen.

## Fazit

Die Ergebnisse der sozialwissenschaftlichen Begleitstudie zeigen, dass die Nutzung von TPR im ambulanten Versorgungssetting nicht allein von technischen Eigenschaften der Systeme abhängt, sondern maßgeblich durch deren Einbettung in soziale und infrastrukturelle Rahmenbedingungen beeinflusst wird. Die Befunde verdeutlichen, dass Akzeptanz und nachhaltige Nutzung insbesondere dort entstehen, wo technische Funktionen mit den Bedürfnissen der Nutzer*innen sowie den Anforderungen des Versorgungskontexts in Einklang stehen. Damit leistet die Studie einen Beitrag zum Verständnis der Bedingungen, unter denen TPR im Alltag tatsächlich genutzt wird und welchen Beitrag sie für eine selbstständige Lebensführung leisten kann. Die Ergebnisse unterstreichen die Notwendigkeit, Implementierungsfragen frühzeitig in die Entwicklung und Einführung entsprechender Technologien einzubeziehen. Neben einer nutzerzentrierten Entwicklung ist daher eine systematische Berücksichtigung des Versorgungskontexts erforderlich. Solange TPR den Anforderungen häuslicher Versorgungssituationen noch nicht ausreichend gerecht werden, können etablierte digitale Lösungen wie Tablets eine praktikable Ergänzung für Pflege- und Therapieanwendungen darstellen.

## Data Availability

Die während der Studie erzeugten und analysierten Datensätze sind aufgrund von Vorgaben des Fördermittelgebers nicht öffentlich zugänglich, können aber auf begründete Anfrage bei der Korrespondenzperson angefordert werden.
